# Pilot safety study of intrabronchial instillation of bone marrow-derived mononuclear cells in patients with silicosis

**DOI:** 10.1186/s12890-015-0061-8

**Published:** 2015-06-11

**Authors:** Marcelo M. Morales, Sérgio A. L. Souza, Luiz Paulo Loivos, Marina A. Lima, Amir Szklo, Leandro Vairo, Taís H. K. Brunswick, Bianca Gutfilen, Miquéias Lopes-Pacheco, Alberto J. Araújo, Alexandre P. Cardoso, Regina C. Goldenberg, Patricia R. M. Rocco, Lea M. B. Fonseca, José R. Lapa e Silva

**Affiliations:** Laboratory of Cellular and Molecular Physiology, Institute of Biophysics Carlos Chagas Filho, da Saude Science Center, Federal University of Rio de Janeiro, Ilha do Fundão, 21941-902 Rio de Janeiro, RJ Brazil; Nuclear Medicine Service, Clementino Fraga Filho University Hospital, Federal University of Rio de Janeiro, Rio de Janeiro, Brazil; Institute of Thoracic Medicine, Clementino Fraga Filho University Hospital, Federal University of Rio de Janeiro, Rio de Janeiro, Brazil; Laboratory of Cellular and Molecular Cardiology, Institute of Biophysics Carlos Chagas Filho, Federal University of Rio de Janeiro, Rio de Janeiro, Brazil; Laboratory of Pulmonary Investigation, Institute of Biophysics Carlos Chagas Filho, Federal University of Rio de Janeiro, Rio de Janeiro, Brazil

**Keywords:** Pneumoconiosis, Chronic inflammation, Lung fibrosis, Cell therapy

## Abstract

**Background:**

Silicosis is an occupational disease for which no effective treatment is currently known. Systemic administration of bone marrow-derived mononuclear cells (BMDMCs) has shown to be safe in lung diseases. However, so far, no studies have analyzed whether bronchoscopic instillation of autologous BMDMCs is a safe route of administration in patients with silicosis.

**Methods:**

We conducted a prospective, non-randomized, single-center longitudinal study in five patients. Inclusion criteria were age 18–50 years, chronic and accelerated silicosis, forced expiratory volume in 1 s <60 % and >40 %, forced vital capacity ≥60 % and arterial oxygen saturation >90 %. The exclusion criteria were smoking, active tuberculosis, neoplasms, autoimmune disorders, heart, liver or renal diseases, or inability to undergo bronchoscopy. BMDMCs were administered through bronchoscopy (2 × 10^7^ cells) into both lungs. Physical examination, laboratory evaluations, quality of life questionnaires, computed tomography of the chest, lung function tests, and perfusion scans were performed before the start of treatment and up to 360 days after BMDMC therapy. Additionally, whole-body and planar scans were evaluated 2 and 24 h after instillation.

**Results:**

No adverse events were observed during and after BMDMC administration. Lung function, quality of life and radiologic features remained stable throughout follow-up. Furthermore, an early increase of perfusion in the base of both lungs was observed and sustained after BMDMC administration.

**Conclusion:**

Administration of BMDMCs through bronchoscopy appears to be feasible and safe in accelerated and chronic silicosis. This pilot study provides a basis for prospective randomized trials to assess the efficacy of this treatment approach.

**Clinical trials.gov identifier:**

NCT01239862 Date of Registration: November 10, 2010

## Background

Silicosis is a pneumoconiosis caused by inhalation of crystalline silica particles. Deposition of these particles in the lung tissue leads to a chronic inflammatory process with formation of silicotic nodules and collagen deposition [1, 2]. To date, there has been no effective therapy to minimize the progression of silicosis [3].

Bone marrow derived-mononuclear cells (BMDMCs) have exhibited beneficial effects for the treatment of various diseases due to their multipotent effects [4]. They are easily obtained for autologous transplantation and can be used on the same day of harvesting at a low cost and without risk of cell rejection (graft-versus-host disease) [5]. Additionally, a preclinical study from our group showed that intratracheal instillation of BMDMCs improved lung mechanics and reduced fibrosis in murine silicosis [6–8].

Based on the foregoing, we tested the hypothesis that intrabronchial instillation through bronchoscopy of autologous BMDMCs in individuals with accelerated and chronic silicosis would be feasible and safe.

## Methods

A prospective, non-randomized, single-center longitudinal study was conducted to evaluate the feasibility and safety of intrabronchial instillation of BMDMCs in patients with accelerated and chronic silicosis. Patients were referred to the Clementino Fraga Filho University Hospital (HUCFF), Federal University of Rio de Janeiro (UFRJ), Brazil, from other institutions. All patients were investigated and treated according to the Occupational Lung Diseases Guidelines for Silicosis of the Brazilian Respiratory Society [9]. The diagnosis of silicosis was established by history of occupational exposure to silica crystals, presence of respiratory symptoms such as shortness of breath and cough, and radiologic abnormalities (bilateral nodular infiltrates with coalescence). The radiographic changes were read and confirmed by at least two independent radiologists. Informed consent was obtained from each patient and the study protocol was approved by the Brazilian National Ethical and Research Committee/CONEP (study ID number: CONEP5772008) and registered at ClinicalTrials.gov (identifier: NCT01239862). In addition to intrabronchial autologous infusion of BMDMCs, the patients received standard care according to the institutional protocol of the Pulmonary Division/HUCFF/UFRJ; however, no patients were treated with inhaled or systemic corticosteroids.

### Study population

Forty-one patients with silicosis were screened for eligibility. All but one were male. The reasons for exclusion were normal spirometry (n = 19), spirometry parameters outside the study range (n = 5), death during screening (n = 3), loss to follow-up during screening (n = 3), refusal to sign informed consent (n = 3), active tuberculosis found at screening (n = 1), patient outside age range (n = 1), and psychiatric disorder (n = 1). Five males were included in the study, with a mean age at intervention of 41 years (range 37–45 years). Patients met the following inclusion criteria: (1) age between 18 and 50 years; (2) chronic or accelerated silicosis characterized by the presence of new areas of fibrosis in high-resolution computed tomography of the lungs in the last 2 years; (3) pulmonary function tests showing moderate to severe impairment, characterized by forced expiratory volume in 1 s (FEV_1_) <60 % predicted and >40 % predicted, and forced vital capacity (FVC) ≥60 % predicted but with arterial oxygen saturation >90 % on room air. Patients were excluded if they met any of the following criteria: (1) unable to undergo bronchoscopy for instillation of cellular material, (2) infectious diseases at the time of the study or 1 month before enrollment, (3) other lung diseases, including active tuberculosis, (4) current or recent smoker, within 12 months of inclusion, characterized by tobacco intake >10 pack-years, (5) intra- or extrathoracic malignant neoplasms, (6) autoimmune disorders, (7) acute or unstable heart failure, (8) acute or uncontrolled coronary insufficiency, (9) primary hematologic disorders, (10) osteopathy reflecting increased risk for bone marrow aspiration, (11) primary or secondary coagulopathies, (12) liver failure, (13) moderate renal failure (creatinine level >2 mg/dL), (14) dependence on respiratory or circulatory support, (15) pregnancy, (16) human immunodeficiency virus seropositivity, and (17) participation in other clinical trials. The primary endpoint focused on safety and included death and respiratory (clinical, radiologic and functional) deterioration.

### Bone marrow aspiration, cell separation and intrabronchial instillation

Bone marrow aspiration and subsequent cell preparation were accomplished on the same day as the infusion of intrabronchial autologous BMDMCs, and lasted 2–4 h. Collection was performed under local anesthesia and light sedation, through puncture and repeated aspirations at the posterior iliac crest region. A total of 80 mL of bone marrow aspirate was collected from each patient, and after removal of bone and fatty residues, mononuclear cells were isolated by a Ficoll-Paque Plus (Amersham Biosciences, São Paulo, Brazil) density gradient, washed three times in saline solution, resuspended in saline with 5 % human albumin, and filtered through a 100-μm nylon filter. After washing, counting, and viability testing, the cells were resuspended in 10 ml saline solution with 5 % autologous serum, and 2 × 10^7^ cells were labeled with 99mTc, as described in previously published protocols [10, 11]. In brief, 500 μl of sterile SnCl_2_ solution was added to the cell suspension in 0.9 % NaCl, and the mixture was incubated for 10 min at room temperature. Then, 45 mCi 99mTc was added and incubation was continued for 10 min. After centrifugation (500 × g for 5 min), the supernatant was removed, the cells were washed again in saline solution, and the pellet was resuspended in saline solution. All cell preparation and labeling procedures were performed in a laminar flow hood. Bacteriological analyses and cultures were also carried out to exclude contamination of the material. A sample of the isolated BMDMCs was characterized by flow-cytometry analysis of specific surface antigens as previously described [11]. The viability of labeled cells was assessed by the trypan blue exclusion test, and was estimated to be greater than 90 % in all cases. A 50-mL aliquot of the autologous BMDMC solution was instilled into each lung and distributed through the lobes by a fiber optic bronchoscope over a 30-min period. Following instillation, patients were positioned in left and right lateral decubitus and Trendelenburg position to facilitate dispersion of the instilled cells. After the procedure, patients were monitored for 1 h and sent to the Nuclear Medicine Department for examinations.

### Flow-cytometry analysis

Isolated BMDMCs were characterized by flow-cytometry analysis of specific surface antigens. Cells were incubated for 20 min at room temperature with primary antibodies conjugated with fluorescein isothiocyanate (FITC), phycoerythrin (PE), allophycocyanin (APC), peridinin-chlorophyll-protein (PercP), and phycoerythrin/cyanine 7 (PE/Cy7). The markers tested included: pan-leukocyte, CD45 (Immunostep); pan T cell, CD3 (BD Biosciences Pharmingen); cytotoxic T cell, CD8 (BD Biosciences Pharmingen); helper T cell, CD4 (BD Biosciences Pharmingen); pan B cell, CD19 (BD Biosciences Pharmingen); NK cell, CD56 (BD Biosciences Pharmingen); promonocyte, CD64 (Immunotech); monocyte, CD14 (IQP); hematopoietic progenitor cells, CD117 (BD Biosciences Pharmingen) and CD34 (BD Biosciences Pharmingen); mesenchymal cells, CD105 (Immunostep), CD73 (BD Biosciences Pharmingen), and CD90 (BD Biosciences Pharmingen); neutrophils, CD31 (BD Biosciences Pharmingen) and CD33 (BD Biosciences Pharmingen); and anti-HLA-DR (MHC-II, BD Biosciences Pharmingen). After staining, erythrocytes were lysed with B&D Lysis Buffer Solution. Data were acquired on a BD FACS CANTO cytometer (BD Biosciences) and analyzed with BD Paint-a-Gate software.

### Measured variables

Patient demographics, medical history, vital signs, routine laboratory tests (blood counts, coagulation tests, biochemical measurements, liver function tests), electrocardiogram, the Saint George Respiratory Questionnaire (SGRQ), the SF-36 Quality of Life Questionnaire, the Modified Borg Dyspnea Scale and the 6-min walk test were assessed using standardized clinical report forms before BMDMC administration (baseline) and at 1, 3, 7, 30, 60, 90, 180, 360 days after the procedure. Lung function tests, including diffusing capacity of lung for carbon monoxide (Dlco) and arterial blood gases, lung CT scans, and lung perfusion scintigraphy were performed at baseline, up to 7 days, and 30, 60, 90, 180 and 360 days after cell transplantation.

### Instillation procedure and imaging analysis

After bone marrow harvesting, approximately 2 × 10^7^ BMDMCs were labeled with technetium-99 m (99mTc) and instilled in the bronchi through fiber optic bronchoscopy. Whole-body, planar and tomographic scintigraphy was carried out at 2 h and 24 h after cell transplantation. All cell preparation and labeling procedures were performed in a laminar flow. Briefly, 500 μL of sterile SnCl_2_ solution was added to the cells and the mixture was incubated at room temperature for 10 min; 45 mCi of 99mTc was then added and incubation continued for another 10 min. After centrifugation (500 × *g* for 5 min), the supernatant was removed and the cells were washed in saline solution. The pellet was also resuspended in saline solution. Viability of the labeled cells was assessed by the Trypan Blue exclusion test, and was estimated to be greater than 93 % in all cases. Labeling efficiency (%) was calculated by the activity in the pellet divided by the sum of the radioactivity in the pellet plus supernatant and was estimated to be greater than 90 % in all cases. Perfusion scintigraphy with 99mTc-MAA (Tc-99 m macroaggregated albumin) was performed before and 30, 60, 120, 180 and 360 days after BMDMC therapy. For regional analysis in both the anterior (A) and posterior (P) images, rectangular regions of interest, equal in size, were drawn over the whole lung (A-whole lung and P-whole lung). Both lungs were divided into six regions of interest: right upper, right middle, right lower, left upper, left middle, and left lower lung fields. Regional blood flow evaluated by 99mTc-MAA perfusion scintigraphy in each region of interest was calculated as per Ohno et al. [12]: Q_PS_ (%) = [(A_roi_ + P_roi_/2)/(A_whole lung_ + P_whole lung_ 2)] × 100, where Q_PS_ is quantitative perfusion scintigraphy, A_roi_ is the anterior region of interest, P_roi_ is the posterior region of interest, A_whole lung_ is the anterior whole lung, and P_whole lung_ is the posterior whole lung.

### Statistical analysis

The Kolmogorov–Smirnov test with Lilliefors’ correction was used to test for normality of data distribution; the Levene median test was used to evaluate the homogeneity of variances. The time course of functional data was evaluated using analysis of variance (ANOVA) followed by Tukey’s test. Differences in quality of life and percentage of perfusion over time were assessed by repeated measures ANOVA on ranks followed by Tukey’s post hoc test. The single imputation method of regression substitution for missing data of two patients at one time point in lung function analysis at 360 days was used. Nonparametric data were analyzed using repeated measures ANOVA on ranks followed by Tukey’s post hoc test. Parametric data are expressed as means ± standard error of the mean; nonparametric data are expressed as the median (interquartile range). The SigmaStat 3.1 statistical software package (Jandel Corporation, San Rafael, CA, USA) was used. A *p*-value <0.05 was considered significant.

## Results

Five patients were included in the study and followed up for 1 year after intrabronchial instillation of autologous BMDMCs. Table 1 presents the patients’ characteristics at entry. All were men, had a mean age at intervention of 41 years (range 37–45 years) and a mean of 10 years (range 3–13 years) of dyspnea on exertion, and were nonsmokers or former-smokers. Exposure to silica was the result of sandblasting (n = 3), quarry truck driving (n = 1), and stone carving (n = 1). Three had a history of tuberculosis treatment in the past, with no activity or sequelae at the time of the study. Lung function findings included a mean post-bronchodilator FEV_1_ of 58.6 % (range, 48.3–64.1 %) and FVC of 87.9 % (61.9–100.1 %), denoting airflow obstruction. The main finding on CT scans was disseminated small nodules and traction bronchiectasis. Sequential CT scans were performed until D360 in all participants, and no major changes were noted compared to the pre-intervention images.

**Table 1:** Demographic and clinical characteristics of the participants

	Patient 1	Patient 2	Patient 3	Patient 4	Patient 5
Age at intervention (years)	43	37	43	45	37
Sex	Male	Male	Male	Male	Male
Race	White	White	White	White	Black
Exposure	Stone carver	Sandblaster	Stone quarry truck driver	Sandblaster	Sandblaster
Duration of symptoms (years)	13	5	3	11	10
Main symptom	Dyspnea on exertion	Progressive dyspnea	Dyspnea on exertion	Dyspnea on exertion	Dyspnea on exertion
Other lung diseases	No	Tuberculosis in 2004	Tuberculosis in 1999	Pneumonia in 2005	Tuberculosis in 2002
Smoking	No	0.6 pack-years, stopped in 2004	8 pack-years, stopped in 1999	No	No
Post-BD FEV_1_ (%)	55.7	48.3	62.1	64.6	62.9
Post-BD FVC (%)	89.3	61.9	94.7	100.1	93.7
Main CT findings at admission	Irregular opacities, mainly at the UULL, with traction bronchiectasis	Diffuse reticulo-nodular infiltrate	Disseminated small nodules, traction bronchiectasis	Disseminated small nodules, traction bronchiectasis	Disseminated small nodules, traction bronchiectasis
Date of infusion	8 Aug 2009	27 Aug 2009	3 Sep 2009	26 Oct 2012	4 Feb 2010
Last outpatient visit and status	3 Mar 2015, stable	3 Dec 2014, stable	3 Dec 2012, stable	22 Nov 2014, stable	22 Aug 2011, active TB^a^

*BD* Bronchodilator, *FEV*_*1*_ Forced expiratory volume in 1 s, *FCV* Forced vital capacity, *CT* Computed tomography, *UULL* Upper lobes, *TB* Tuberculosis

^a^moved to another state

Bronchoscopy and administration of BMDMCs were well tolerated. Two patients experienced mild wheezing after the infusion, which resolved quickly with bronchodilator nebulization. No immediate respiratory, cardiovascular, or hematological complications or other adverse events were observed throughout the 1-year follow-up.

**Fig. 1:**
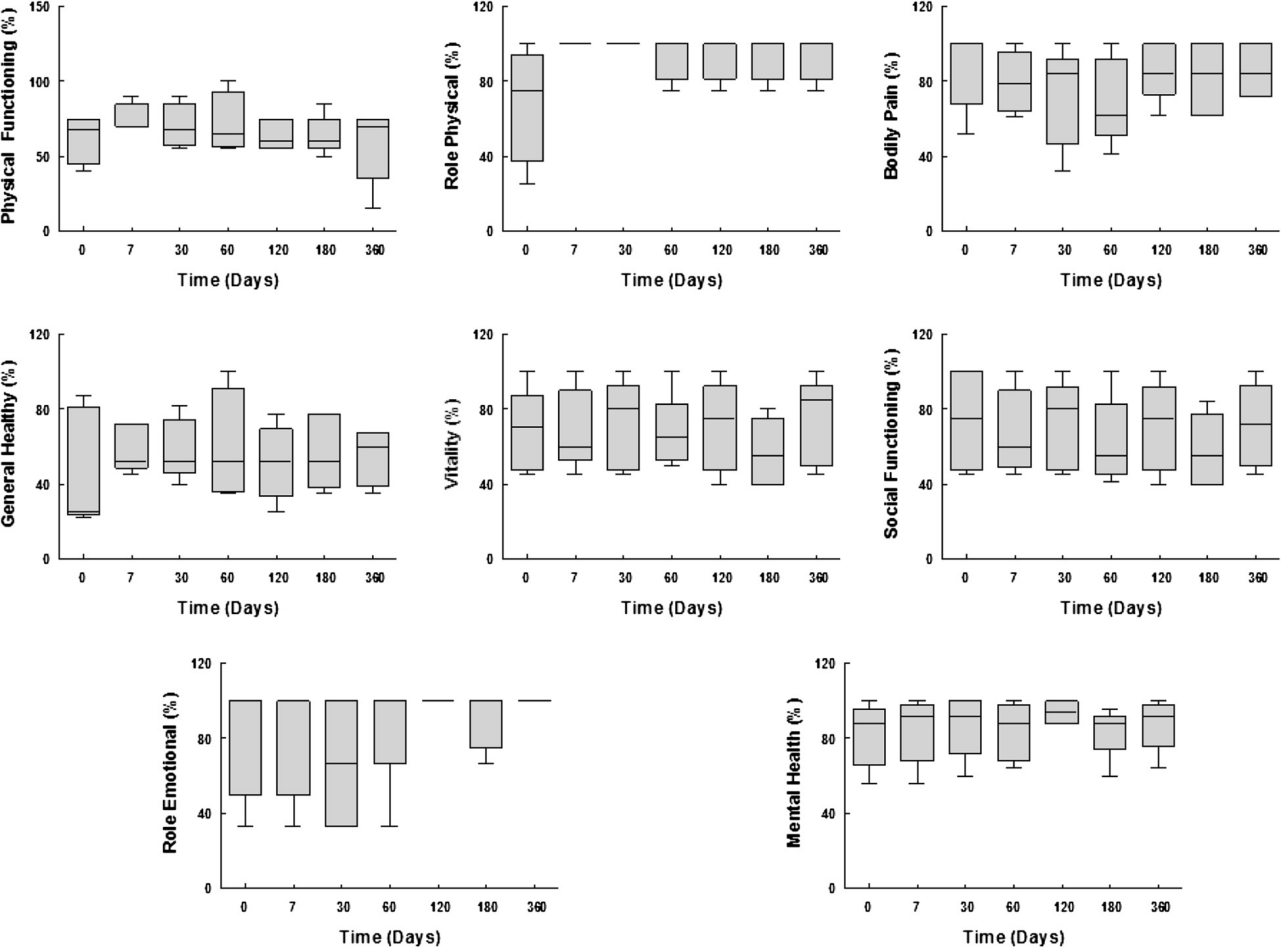
Evolution of quality of life domains (physical functioning, role-physical, bodily pain, general health, vitality, social functioning, role-emotional, mental health), as measured by the SF-36 questionnaire, during the 360 days follow-up period. All parameters remained stable throughout the observation period. Values are median (25th–75th percentile) of 5 patients at each time point

Table 2 describes the progression of respiratory function during the study. After the first month, FEV_1_ improved in the first patient, with no further changes in other patients. Figure 1 illustrates the progression of quality of life domains during the follow-up period, as measured by the SF-36 questionnaire. These parameters remained stable throughout the observation period.

**Table 2:** Functional parameters

	Day 0	Day 7	Day 30	Day 60	Day 180	Day 360
FVC (%)	88.0 ± 15.1	86.3 ± 15.5	87.0 ± 17.2	86.1 ± 14.1	83.8 ± 14.5	88.0 ± 22.8
FEV_1_ (%)	58.7 ± 6.7	56.9 ± 5.4	57.9 ± 5.6	57.6 ± 4.8	55.6 ± 6.6	55.5 ± 7.2
TLC (%)	87.1 ± 17.3	87.5 ± 18.0	86.9 ± 23.0	86.3 ± 18.7	87.8 ± 18.4	84.5 ± 20.5
Dlco (%)	60.0 ± 16.4	60.2 ± 16.5	55.8 ± 17.9	58.5 ± 14.3	56.0 ± 17.0	57.5 ± 5.0

Data presented as mean ± standard deviation of 5 silicotic patients

*FEV*_*1*_ Forced expiratory volume in 1 s, *FCV* Forced vital capacity, *TLC* Total lung capacity, *TLC* Total lung capacity, *D* *lco* Diffusing capacity of the lung for carbon monoxide

**Fig. 2:**
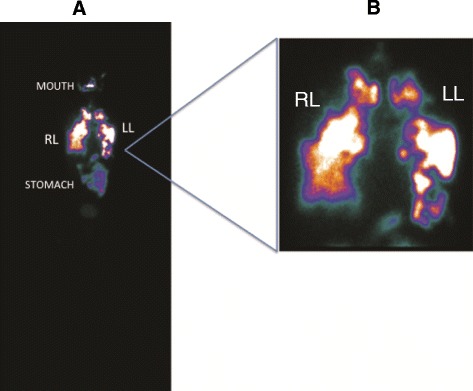
**a** Representative image of whole-body scintigraphy showing the biodistribution of 99mTc-labeled BMDMCs 2 h after instillation in one patient; stem cells are seen mainly in the lungs. **b** There is also uptake in the mouth and stomach, due to minor swallowing during instillation. RL, right lung; LL, left lung; BMDMCs, bone marrow–derived mononuclear cells; 99mTc-BMDMCs, 99mTc-labeled bone marrow–derived mononuclear cells

**Fig. 3:**
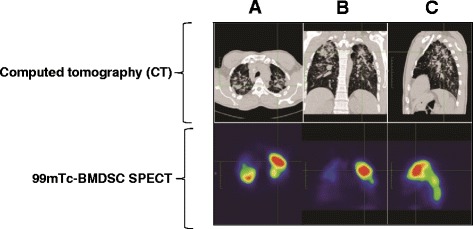
Representative image of CT and 99mTc-BMDSC SPECT showing correspondence of a left lung lesion (green triangular area). CT, computed tomography; 99mTc-BMDSC, 99mTc-labeled bone marrow–derived mononuclear cells; SPECT, single-photon emission computed tomography

**Fig. 4:**
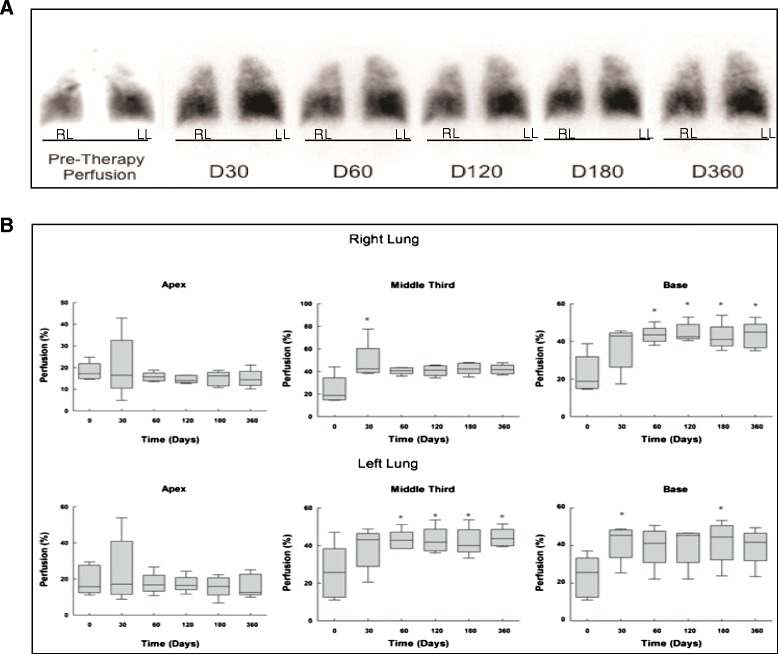
**a** 99mTc-macroaggregated albumin perfusion scintigraphy sequences from before cell therapy until 360 days after cell therapy, showing normal uptake at the right base and decreased uptake at both apexes. Improvement in the left base can be seen from day 120 to day 360. **b** Values are median (25th–75th percentile) of 5 patients at each time point. Statistical analysis of lung perfusion, showing significant differences mainly in the base of the right lungs from day 30 to day 360 as compared with baseline. RL, right lung; LL, left lung

Imaging findings are shown in Figs. 2, 3 and 4. Figure 2a is a representative image of the heterogeneous distribution of the labeled cells in both lungs 2 h after instillation, with some uptake in the mouth and stomach due to swallowing of the instilled cells during bronchoscopy. Cells were identified in the lungs 24 h after instillation, with a similar distribution as at the 2-h time point (Fig. 2b). Figure 3 illustrates that cells were located mainly at sites exhibiting advanced fibrotic damage in the lungs.

Follow-up lung perfusion findings are illustrated in Fig. 4. Statistical analysis demonstrated that percent perfusion at the apex of the right and left lungs did not differ during the study period. In the middle third, percent perfusion was increased at day 30 in the right lung, whereas in the left lung it was higher at day 60 and remained unaltered at days 120, 180, and 360. In the base of the right lung, percent perfusion was higher at days 60, 120, 180, and 360 compared with baseline, whereas a significant difference was observed at days 30 and 180 in the left lung (Fig. 4).

## Discussion

The present study evaluated the safety of autologous BMDMC administration through bronchoscopy in patients with silicosis over a period of 360 days. To the best of our knowledge, this was the first study to evaluate the safety of autologous BMDMCs in patients with silicosis. The procedure was well tolerated and no adverse events were observed in the follow-up period. Even though no significant changes were reported in lung function and quality of life, autologous BMDMC transplantation seems to have led to an early increase in perfusion at the base of both lungs, which remained increased for the duration of the follow-up.

Recent studies have investigated the impact of either autologous BMDMCs or mesenchymal stem cells (MSCs) on lung diseases [13–16]. Both cells present particular advantages: BMDMCs can be used in autologous transplantation on the same day of harvesting, avoiding common complications such as graft-versus-host disease, whereas MSCs have multilineage differentiation potential and immune-privileged features that enable allogenic use. So far, clinical studies have demonstrated the safety of systemic BMDMC/MSC infusion in lung diseases, with no early or late adverse effects reported [13]. Intravenous infusion is often used in preclinical and clinical studies for the delivery of various cell types, since this route of administration provides broader biodistribution and is easy to perform [17]. However, the administration of BMDMCs through bronchoscopy into different areas of the lungs may result in a greater number of cells at the site of injury [18, 19]. We administered a fixed amount of BMDMCs (2 × 10^7^), independent of body weight, based on preclinical and clinical studies on different diseases [10, 11, 20]. To evaluate the early distribution of the injected cells into the lungs, cells were labelled with 99mTc on the basis of previously published protocols [21, 22]. *In vivo* imaging and quantification of stem cells is an essential tool for stem cell tracking, although it has inherent limitations. For instance, regardless of which technique is used, cell labeling may cause cellular damage due to the labeling chemicals, and should be carefully controlled. Due to the short half-life of 99mTc (approximately 6 h), we cannot rule out that the amount of cells in the lungs may increase at later time points. Other radiopharmaceutical compounds such as indium-111 oxine would allow monitoring for up to 96 h, but have disadvantages, such as the interval of 18–24 h between infusion and imaging that is usually necessary, suboptimal photon energy, and a higher radiation burden for the cells and for the patient [23]. We also used 99mTc-MAA perfusion scintigraphy to follow the likely pattern of distribution of the cells for up to 360 days after infusion.

No adverse events or deaths occurred among the patients treated with BMDMCs. Clinical, functional and radiological parameters were evaluated at 1-year follow-up. The clinical features of this group were stable throughout the follow-up period and no clinical signs or symptoms of bronchitis or pneumonia were observed after BMDMC administration. Even though a 1-year period is not long enough to allow definitive conclusions, it is well known that lung function deteriorates more quickly in patients with silicosis depending on continuation of exposure to silica dust, age and grade of lung lesions [23–25]. The volunteers in our study were relatively young with moderate lung lesions, and exhibited clear stabilization of lung function, which could be attributable to the intervention. This hypothesis would be more clearly demonstrated if a control group with similar characteristics was added to the study. With regard to the quality of life questionnaires, no changes were seen after 360 days of follow-up. An early increase in perfusion at the base of both lungs was observed and sustained after BMDMC administration. Based on CT scan analysis, fibrosis was not prominent in the apex of the lung, but distributed across different lung segments. Therefore, the increased perfusion observed in both lung bases was likely not associated with a predominance of fibrosis in any specific segment of the lung. In line with this observation and based on our preclinical data showing that BMDMCs reduce fibrosis in a mouse model of silicosis, we may hypothesize that these cells mitigated progression of fibrosis through paracrine effects.

Since this is a safety study, analysis was only observational, and further larger-scale trials are necessary to fully examine the efficacy of BMDMCs in patients with silicosis.

Experimental studies have demonstrated the efficacy of systemic and intratracheal administration of BMDMCs in a murine model of silicosis [6–8, 26] that resembles human silicosis. BMDMC therapy (intratracheally or intravenously administered) reduced both lung inflammation and remodeling, thus improving lung mechanics, through paracrine signaling [6–8, 26]. Conversely, in the present study, no significant changes in lung function or quality of life indicators were observed over 360 days of analysis. The fact that the clinical result did not reflect the experimental data may be associated with differences in the course of the disease between animal models and humans, the number of cells administered, and the timing of analysis.

Our pilot study was limited by its small sample size; however, it was the first investigation to evaluate stem-cell therapy in silicotic patients. Further studies should be performed in a greater number of patients, with the addition of a control group, and in patients with mild and severe silicosis. Clinical trials evaluating the effects of MSC therapy in idiopathic lung fibrosis (ILF) have been published [15, 16]. However, this was the first study that analyzed the safety of autologous BMDMCs in patients with silicosis and, in contrast with the studies, no adverse events were observed. In this line, two clinical trials involving the endobronchial or systemic delivery of autologous adipose tissue-derived [15] or placenta-derived mesenchymal stem cells [16] demonstrated that these treatments are not safe in patients with IPF. In the first clinical trial, transient fever (50 % of patients), cough, dyspnea, and increased heart rate (14 % of patients) were observed following each endobronchial cell administration [15], and one patient experienced a change in absolute FVC of more than 10 % at 6 months, suggesting disease progression. A second phase 1b study on the use of exogenous placenta-derived MSCs in patients with IPF also observed adverse effects after therapy [16].

### Future perspectives

Clinical studies are required to evaluate the safety of different doses and treatment intervals of BMDMC administration and to analyze the efficacy of this approach, including pulmonary and systemic hemodynamics, lung function, hospitalizations, infections, and death. The use of a single infusion or repeated infusions also needs to be addressed, even though multiple infusions could lead to right heart overload (and pulmonary hypertension) [27]. Most patients with silicosis are not diagnosed in the early stages of the disease, but identification of these patients should be considered for a clinical trial of cell therapy. Clinical trials should include patients with different degrees of lung function impairment due to silicosis.

## Conclusion

Based on this preliminary study, the administration of BMDMCs through bronchoscopy appears to be safe. Certainly, further clinical investigation of BMDMCs or even other cell types in patients with silicosis is warranted.
